# ALDH1/2 deficiency modulates dieldrin-induced dopaminergic neurodegeneration and reactive glial morphometry in male mice

**DOI:** 10.3389/ftox.2026.1888799

**Published:** 2026-09-15

**Authors:** Omar A. Yanouri, Adam J. Schuller, Savannah M. Rocha, Aidan M. Briggs, Paul A. Martinez, Elizabeth Fernandez, Randy Strong, Xueshu Li, Hans-Joachim Lehmler, Jonathan A. Doorn, Ronald B. Tjalkens

**Affiliations:** 1 Department of Environmental and Radiological Health Sciences, Colorado State University, Fort Collins, CO, United States; 2 Department of Neurology, Barrow Neurological Institute, Phoenix, AZ, United States; 3 Department of Pharmacology, Barshop Institute, University of Texas San Antonio Health Sciences Center, San Antonio, TX, United States; 4 Geriatric Research, Educational and Clinical Center (GRECC), South Texas Healthcare System, San Antonio, TX, United States; 5 Department of Occupational and Environmental Health, College of Public Health, University of Iowa, Iowa City, IA, United States; 6 Department of Pharmaceutical Sciences and Experimental Therapeutics, College of Pharmacy, University of Iowa, Iowa City, IA, United States

**Keywords:** dieldrin, microglia, neurodegeneration, organochlorine, Parkinson’s disease, pesticide

## Abstract

The etiology of Parkinson’s disease (PD) is associated with exposure to environmental contaminants, including the organochlorine pesticide dieldrin. This is thought to occur through induction of oxidative damage and dysregulation of dopamine homeostasis via impairment of aldehyde dehydrogenase (ALDH) enzymes, resulting in neuronal stress that may contribute to glial-mediated neurotoxicity. We postulated that sub-chronic murine dieldrin exposure would induce PD-relevant histopathologic changes in the substantia nigra (SN) which would be exacerbated in ALDH-deficient mice. To test this hypothesis, six-month-old wildtype and Aldh1a1^−/−^ X Aldh2^−/−^ (Aldh1/2 KO) mice were fed dieldrin in chow at doses of 0, 0.2, or 0.6ppm for 6 weeks. Slide scanning microscopy and deep learning-based image analysis were then applied to quantify neuronal injury and glial morphometry. Dieldrin exposure resulted in male-specific dopaminergic neurodegeneration, a dose-dependent increase in astrocytic density, and disease-associated alterations in microglial morphology in the substantia nigra. Interestingly, Aldh1/2 KO attenuated neuronal loss and distinctly modulated reactive astrocytosis and microglial morphometric changes. These results highlight a sex-dependent susceptibility to dieldrin-induced neurotoxicity and suggest a role of ALDH in modulating glial reactivity in the context of PD-like neurodegeneration.

## 1 Introduction

Parkinson’s disease (PD) is the fastest growing neurodegenerative disease (Global Burden of Disease Results, 2024; Collaborators, 2024; Su et al., 2025) and is characterized by the loss of dopaminergic neurons in the substantia nigra pars compacta (SNpc), as well as inflammatory activation of microglia and astrocytes (Kam et al., 2020; Surmeier et al., 2017; Spillantini et al., 1997). Over 90% of PD cases are idiopathic, although exposure to environmental toxicants has been strongly associated with increased risk for onset (Ball et al., 2019; Dirandeh et al., 2022; Pouchieu et al., 2018; Willis et al., 2022; Pang et al., 2019). Dieldrin is one such contaminant, with a history of extensive use as an agricultural pest control agent (Jorgenson, , 2001; Honeycutt et al., 2014). Elevated levels of dieldrin have been observed in the serum and brain of PD patients (Chhillar et al., 2013; Corrigan et al., 2000) and epidemiologic studies have suggested an association between dieldrin exposure and PD risk (Weisskopf et al., 2010).

The mechanism underlying dieldrin-induced neurodegeneration remains unclear. *In vitro* studies have demonstrated ability of this toxicant to disrupt dopamine (DA) metabolism, induce reactive oxygen species (ROS) generation, and disrupt mitochondrial function (Weisskopf et al., 2010; Sanchez-Ramos et al., 1998; Allen et al., 2013; Kitazawa et al., 2003; Kitazawa et al., 2001; Schmidt et al., 2017). Glial cells respond to these signals, which may indicate neuronal damage, by adopting reactive phenotypes (Bohatschek et al., 2001; Chang et al., 2024). A growing body of work reports elevated biomarkers of neuroinflammation (Fitzmaurice et al., 2013; Mogi et al., 2007) as well as aberrant glial proliferation and morphology in PD patient brains (Imamura et al., 2003; Mazumder et al., 2022). Our group has reported similar inflammatory activation of microglia and astrocytes across a variety of toxicant-based murine models of PD (Rocha et al., 2022; Kirkley et al., 2017). However, limited data exist examining glial reactivity in response to dieldrin. Further, recent data indicate that this toxicant inhibits aldehyde dehydrogenase (ALDH), potentially exacerbating PD-like neurotoxicity through the accumulation of reactive DA metabolites (Allen et al., 2013; Fitzmaurice et al., 2014).

Together, these data motivated the present assessment of PD-relevant histopathology following dieldrin exposure in both wildtype (WT) and genetically ablated Aldh1a1 and Aldh2 (Aldh1/2 KO) mice. We postulated that sub-chronic exposure to this toxicant would result in disease-associated changes in glial morphology as well as nigrostriatal dopaminergic neurodegeneration. We further hypothesized that Aldh1/2 KO would exacerbate the deleterious effects of dieldrin exposure in this context. To interrogate these hypotheses, WT and Aldh1/2 KO mice were exposed to dieldrin via chow over 6 weeks. Slide-scanning microscopy and deep learning-based image analysis were employed to quantify nigrostriatal dopaminergic neurodegeneration and glial reactivity. These cutting-edge approaches in digital pathology allowed us to interrogate the cellular changes underlying the neuropathology of this toxicant at scale.

## 2 Materials and methods

### 2.1 Animal husbandry and dosing

All experiments involving animals were conducted with approval of the Institutional Animal Care and Use Committee at the University of Texas Health Science Center, the South Texas Veterans Healthcare System (San Antonio, TX, United States). Six-month-old male and female WT and Aldh1/2 KO mice, previously characterized (Wey et al., 2012; Fernandez et al., 2006), were housed by sex (<6/cage) in a ventilated cage system with paper bedding on a 12-h light-dark cycle at 23 °C–25 °C with ad libitum food and water. WT and KO mice were fed dieldrin formulated in NIH-07 chow (Dyets, Inc., Bethlehem, PA) at 0, 0.2, or 0.6 ppm for 6 weeks.

### 2.2 Tissue processing

Mice were euthanized via CO_2_ inhalation followed by decapitation. Fresh tissue was flash-frozen and stored at −80 °C until use. Fixed brains were submerged in 4% paraformaldehyde before processing by Colorado State University’s Veterinary Diagnostic Laboratory. Embedded tissue was coronally sectioned at 5 µm thickness onto charged slides.

### 2.3 Mass spectrometry analysis

#### 2.3.1 Dieldrin extraction from mice brain

Dieldrin was extracted from striatal tissue samples via a published liquid-liquid extraction method protocol (Li et al., 2022). Briefly, samples (13 ± 3 mg, n = 45) were homogenized for 30 s in isopropanol. Method blanks and ongoing precision and recovery (OPR) samples were processed in parallel. 1 mL of diethyl ether and surrogate standard endrin were added to each sample and dieldrin was spiked into OPR samples. Tubes were inverted for 5 min, then centrifuged at 1,690 g for 5 min. The supernatant was transferred to a new glass tube. Tissue residue was re-extracted with isopropanol and hexane:diethyl ether (9:1, v/v). These extracts were washed with 1% KCl solution, and re-extracted. The product was concentrated via solvent evaporation system. An internal standard PCB204 and hexane were added. The mixture was vortexed and transferred to autosampler vials and stored at – 20 °C until GC-MS analysis.

#### 2.3.2 Gas chromatographic determination

Samples were analyzed using an Agilent 7890A gas chromatograph coupled with an Agilent 5975C Inert Mass Selective Detector (MSD) operated in electronic ionization mode. Samples were analyzed on SLB-5MS capillary column (30 m length, 250 μm inner diameter, 0.25 μm film thickness; Supelco, Bellefonte, PA, United States) in the selected ion monitoring (SIM) mode. The temperature program was 50 °C, held for 1 min, 10 °C/min to 150 °C, then 5 °C/min to 280 °C, and held for 10 min (Li et al., 2020). The injector temperature was 280 °C, and the operating temperatures were 280 °C, 230 °C and 150 °C for transfer line, source, and quadrupole, respectively. The flow rate of carrier gas helium was 1 mL/min.

#### 2.3.3 Quality assurance and quality control

A series of concentrations of from 1 to 1,000 ng/mL demonstrated linearity (R^2^ > 0.999). The limit of detection (LOD, ng/g) was calculated following EPA formula, LOD = mean_control_ + t_0.99, n-1_ × SD_control_, where mean_control_ represents the average tissue weight-adjusted values of determined in tissue blank samples, SD_control_ indicates the standard deviation of tissue blanks, and t_0.99, n-1_ is the students’ t value at the 99% confidence level with n-1 degree of freedom. The method detection limit (MDL, ng) was calculated similar to LOD using method blank samples. The MDL and LOD for dieldrin are 0.88 ng (n = 7), and 43.41 ng/g (n = 9), respectively. The recovery rates of surrogate standard across all the samples were 88% ± 11% (n = 62), with relative standard deviation of 13%. The recovery rates of dieldrin in OPR samples were 89% ± 8% (n = 3).

### 2.4 Immunoblotting

Striatal tissue was homogenized in protein isolation buffer (AM-1921, ThermoFisher) supplemented with protease and phosphatase inhibitors (PI78440, ThermoFisher). Protein concentration was quantified via BCA (23,235, ThermoFisher) and samples were denatured in lithium dodecyl sulfate sample buffer (NP0009, Invitrogen). 10–20 μg of protein were separated on a 4%–20% Bis-Tris-SDS-PAGE gel (Biorad, 5671095) in MES buffer and transferred to a PVDF membrane. Membranes were probed overnight at 4 °C against the following primary antibodies: mouse anti-GFAP (1:1,000; sc-33673, Santa Cruz), mouse anti-TNF-α at (1:1,000; sc-52746, Santa Cruz), mouse anti-IFN-γ (1:1,000; sc-74106, Santa Cruz), and mouse anti-GAPDH (1:5000; 60004-1-Ig, Proteintech). LiCOR Odyssey imaging system (LiCOR, Lincoln, NE) and analyzed age Studio Lite software (LiCOR) were utilized to visualize and analyze the immunoblots, respectively.

### 2.5 Histopathological staining

Brain sections were deparaffinized and immunofluorescently labeled using either a Leica Bond RXm or ONCORE Pro X automated slide staining system (Leica Biosystems, Nussloch GmbH; Biocare Medical, Pacheco, CA, United States). For sections stained via the Leica RXm staining robot, antigen retrieval was performed using heated Bond Epitope Retrieval Solution. Sections were permeabilized in 0.1% Triton X and blocked in 1% donkey and goat serum. Primary antibodies were incubated for 1 h at room temperature (RT) including: rabbit anti-tyrosine hydroxylase (TH) (1:500; Millipore AB152) and goat anti-Iba1 (1:50, Abcam ab5076). Species-specific secondary antibodies were applied, followed by nuclear counterstaining with Hoechst 33,342 (1:5,000, Invitrogen H3570). For staining performed on the ONCORE Pro X, antigen retrieval was performed using heated Antigen Retrieval 1/2 (Biocare Medical) buffers. Primary antibodies included: rabbit anti-GFAP (1:1,000, Abcam ab7260) and rabbit anti-TH (1:500, Millipore AB152) diluted in 1X Akoya Antibody Diluent. Primary antibodies were sequentially detected with MACH 2 Rabbit HRP-Polymer (Biocare Medical RHRP520) via Opal 570 (Akoya FP1488001KT) and Opal 620 (Akoya FP1495001KT) diluted 1:250 in 1X Akoya Amplification Buffer. Sections were counterstained with spectral DAPI (1:20) to identify nuclei. All slides were coverslipped using ProLong Gold Antifade hard set mounting medium. Hematoxylin and eosin (H&E) staining was performed by Colorado State University’s Veterinary Diagnostic Laboratory.

### 2.6 Microscopy

Microscopy was conducted using an Olympus VS200 microscope (Olympus Corporation, Tokyo, Japan) equipped with a Hamamatsu ORCA-Fusion-LT CCD camera (Hamamatsu Photonics, Shizuoka, Japan). Montage images of TH^+^ neurons, GFAP^+^ astrocytes, and Iba1^+^ microglia residing in the SN were collected via Olympus UPLXAPO20X (0.8 N.A.) objective. Additionally, brightfield microscopy was utilized to acquire images of H&E-labeled sections spanning the SN using an Olympus UPLXAPO40X (0.95 N.A.) objective. All images were obtained and analyzed under the same conditions for exposure time, light source intensity, magnification, camera gain, and filter application per channel.

### 2.7 Stereological assessment of neuronal numbers

Unbiased stereological quantification of neurons was adapted from previously reported methods (Rocha et al., 2022; Bantle et al., 2019; Rocha et al., 2023; Tapias and Greenamyre, 2014). A 1:24 sampling fraction was used, resulting in 4–6 sections per animal spanning the SNpc. Regions of interest (ROIs) were manually annotated per standard anatomical landmarks from the murine Allen Brain Atlas, using Visiopharm Oncotopix (v2024.07). Deep learning-based neural networks were trained on TH^+^/DAPI^+^ neuronal nuclei in the SNpc across all experimental groups to approximately 100,000 iterations with a loss function of less than 0.05. Efficacy was manually assessed every 20,000 iterations. Separate neural networks generated to identify H&E-stained neurons were trained to identify non-eosinophilic, intact neuronal nuclei.

### 2.8 Quantification of striatal tyrosine hydroxylase intensity

Two sections per animal encompassing the rostro-caudal extent of the caudate and putamen were selected based on anatomical region. Quantitative analysis was performed on montage scans of fluorescently TH-labeled sections according to previously published methods (Rocha et al., 2023). Briefly, mean TH intensity normalized to background autofluorescence was ascertained within ROIs.

### 2.9 Assessment of glial density and morphometry within the substantia nigra

Characterization of SN glial cells was adapted from previously reported methods (Hager et al., 2026). Briefly, 1–2 sections per animal within the SN were selected for fluorescent labeling with markers for microglia (Iba1) and astrocytes (GFAP), followed by montage imaging. ROIs encompassing the substantia nigra pars compacta and pars reticulata were manually annotated on captured images via the Allen Brain Atlas. Iba1^+^/DAPI^+^ and GFAP^+^/DAPI^+^ cells, as well as GFAP^+^ area, were detected using custom-trained deep learning-based neural networks in Visiopharm Oncotopix. Networks were separately trained to approximately 150,000 iterations with a loss function of less than 0.05 on Iba1^+^/DAPI^+^ and GFAP^+^/DAPI^+^ of SN across all experimental groups. Efficacy was manually assessed every 20,000 iterations. Probability maps were generated by adjusting band features allowing for pixel-wide skeletonization of glial arbors. Assessment of glial morphometric parameters included: sum of process lengths/cell, volume/cell, number of branches/cell, and number of processes/cell. Additional parameters were gathered for dimensional reduction analysis via generation of Uniform Manifold Approximation and Projection (UMAP) plots using the umap (v0.2.10.0) package in R (v 4.5.2) utilizing a Euclidean distance metric. These included: sum of process lengths/cell, volume/cell, log-transformed number of branches/cell, log-transformed number of processes/cell, convexivity, ellipticalness, form factor, perimeter, eccentricity, solidity, Iba1 intensity, DAPI intensity, and nuclear area.

### 2.10 Statistical analysis

Data are presented as mean ± SEM, unless otherwise noted. Power analysis was performed using G*Power (v3.1.9.5), for which an N of 64 (n > 5/group) was determined for an effect size of 0.4, type-I error of 0.05, and Power of 0.8. Experimental values for each group were analyzed with a ROUT outlier analysis (Q = 0.01) in GraphPad Prism (v10.4.2) or median average deviation (threshold = 3) in RStudio (v2026.01.0; R v4.5.2) via the Routliers package (v0.0.0.3) to identify significant outliers for removal (<5% of all datapoints excluded). Differences between sex, genotype, and treatment conditions were analyzed using two- and three-way ANOVAs with Bonferroni’s post-hoc testing. Glial morphology was analyzed using Aligned ranks transformation ANOVA and negative-binominal general linear models using the ARTools (v0.11.2) and MASS (v7.3–65) packages, respectively, with Bonferroni’s post-hoc testing applied utilizing the emmeans (v2.0.1) package. UMAP plots were generated via the umap (v0.2.10.0) package. Significance i identified as *p < 0.05, **p < 0.01, ***p < 0.001, and ****p < 0.0001.

## 3 Results

### 3.1 Aldehyde dehydrogenase 1/2 dual knockout protects against dopaminergic neurodegeneration in the substantia nigra pars compacta

Mice were fed dieldrin in chow at 0, 0.2, or 0.6 ppm for 6 weeks (n = 5–6 mice/genotype/group). Dieldrin levels in the striatum were assessed via GC-MS (Supplementary Table 1). Dieldrin concentrations were detectable in a portion of samples from the low-dose (LD) 0.2ppm condition (Male WT: 79.02 ± 4.49 ng/g; female WT: 77.29 ng/g; male KO: 74.58 ng/g; female KO: 69.94 ng/g), and in all samples in the high-dose (HD) 0.6ppm condition (male WT: 104.8 ng/g; female WT: 185.2 ng/g; male KO: 130.3 ng/g; female KO: 124.0 ng/g). No differences were observed in body weight by dieldrin exposure across any of the groups; however, a significant main effect of genotype was observed for change in body weight of male (p = 0.0001) and female (p < 0.0001) mice (Supplementary Figure S1), which is consistent with prior work demonstrating Aldh inhibition may limit weight gain in rodents (Haenisch et al., 2018; Kiefer et al., 2012). To determine the effect of this exposure to PD-relevant pathology, we quantified the number of dopaminergic neurons across the SNpc using the motorized stage stereology method, as previously reported (Rocha et al., 2023; Tapias and Greenamyre, 2014) (Figure 1). This revealed a male-specific reduction of 16.20% (p = 0.0004) among HD WT mice (Figures 1A–C,M). No change was noted for male Aldh1/2 KO mice, nor female mice of either genotype (Figures 1D–L). Concurrently, quantification of neuronal soma in the SNpc was performed on H&E-stained sections (Supplementary Figure S2). Male WT mice demonstrated a dose-dependent reduction in healthy neurons of 26.83% and 38.68% (p < 0.0001) in the LD and HD conditions, respectively (Figure 1N). There did appear to be a qualitative difference in the prevalence of pyknotic-like neuronal nuclei between HD male WT and KO mice (Supplementary Figure S2). No change in the density of TH^+^ terminals in the striatum was detected across genotype or treatment condition (Supplementary Figure S3). However, a main effect of dose (p = 0.0050) and interaction effect of sex and genotype (p = 0.0302) was identified.

**FIGURE 1:**
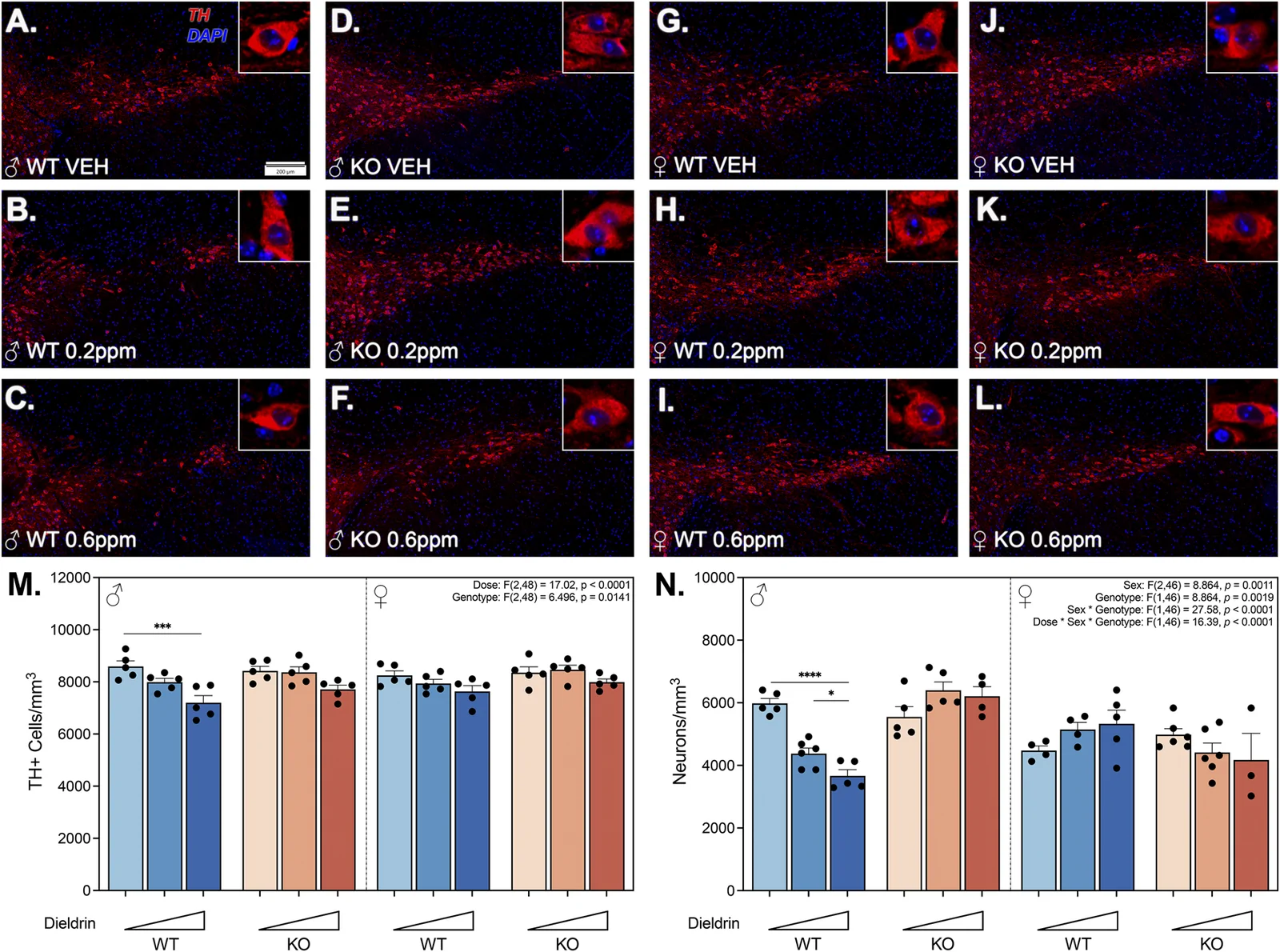
Sub-chronic dieldrin exposure results in male-specific neurodegeneration in the SN. Representative montage images **(A–L)** of the SN with immunofluorescent labeling of dopaminergic neurons (TH, red) and nuclei (DAPI, blue); scale bar 200 µm. Bar graphs depict the results of stereologic quantification of dopaminergic (TH+/DAPI+; n = 5–6 mice/group) **(M)** and total neuronal density **(N)** (n = 3–5 mice/group) within the SNpc. Statistical analysis was conducted using parametric three-way ANOVAs with Bonferroni post-hoc correction. Significant main and interaction effects are denoted in the upper right-hand corner of each graph. Data are expressed as mean ± SEM. Dieldrin doses utilized are 0 ppm, 0.2 ppm, and 0.6 ppm. *p < 0.05; p*** <0.001; ****p < 0.0001.

### 3.2 Dieldrin exposure results in dose-dependent reactive astrocytosis

Following the assessment of neuronal loss, we sought to interrogate glial reactivity in the SN. As dopaminergic neurotoxicity was observed in a sex-dependent manner, examination of astrocytes was performed only in male mice. A genotype-specific dose-response relationship was observed in astrocytic density, with HD WT mice demonstrating an 81.76% (p = 0.0027) increase in GFAP^+^/DAPI^+^ cells relative to control samples, and a difference of 83.71% (p = 0.0010) as compared to KO counterparts (Figures 2A–E). WT mice in the HD group displayed a reduction in nigral GFAP^+^ area 50.81% (p = 0.0294; Figure 2F). Separately, assessment of the total astrocyte territory revealed a 14.15% (p = 0.0277) decrease in LD WT mice from control, in addition to a difference of 18.07% (p = 0.0002) and 23.89% (p < 0.0001) relative to vehicle-normalized LD and HD KO counterparts, respectively (Figure 2G). Concurrently, WT HD astrocytes showed a 19.13% reduction in total process length as compared with control (Figure 2H). To further interrogate these morphometric changes, we examined the ratio of astrocyte area/branch count, and total process length/branch count. A dose-dependent increase in volume/branches ratio was observed for WT mice in the LD (1.70%, p = 0.0257) and HD (14.63%, p = 0.0051) conditions (Figure 2I). These changes were greater than that observed in KO counterparts (LD: 9.16%, p = 0.0004; HD: 15.08%, p = 0.0001). The length/branches ratio differed across genotype, with a difference of 4.42% (p = 0.0070) and 5.02% (p = 0.0409) across LD and HD conditions, respectively (Figure 2J).

**FIGURE 2:**
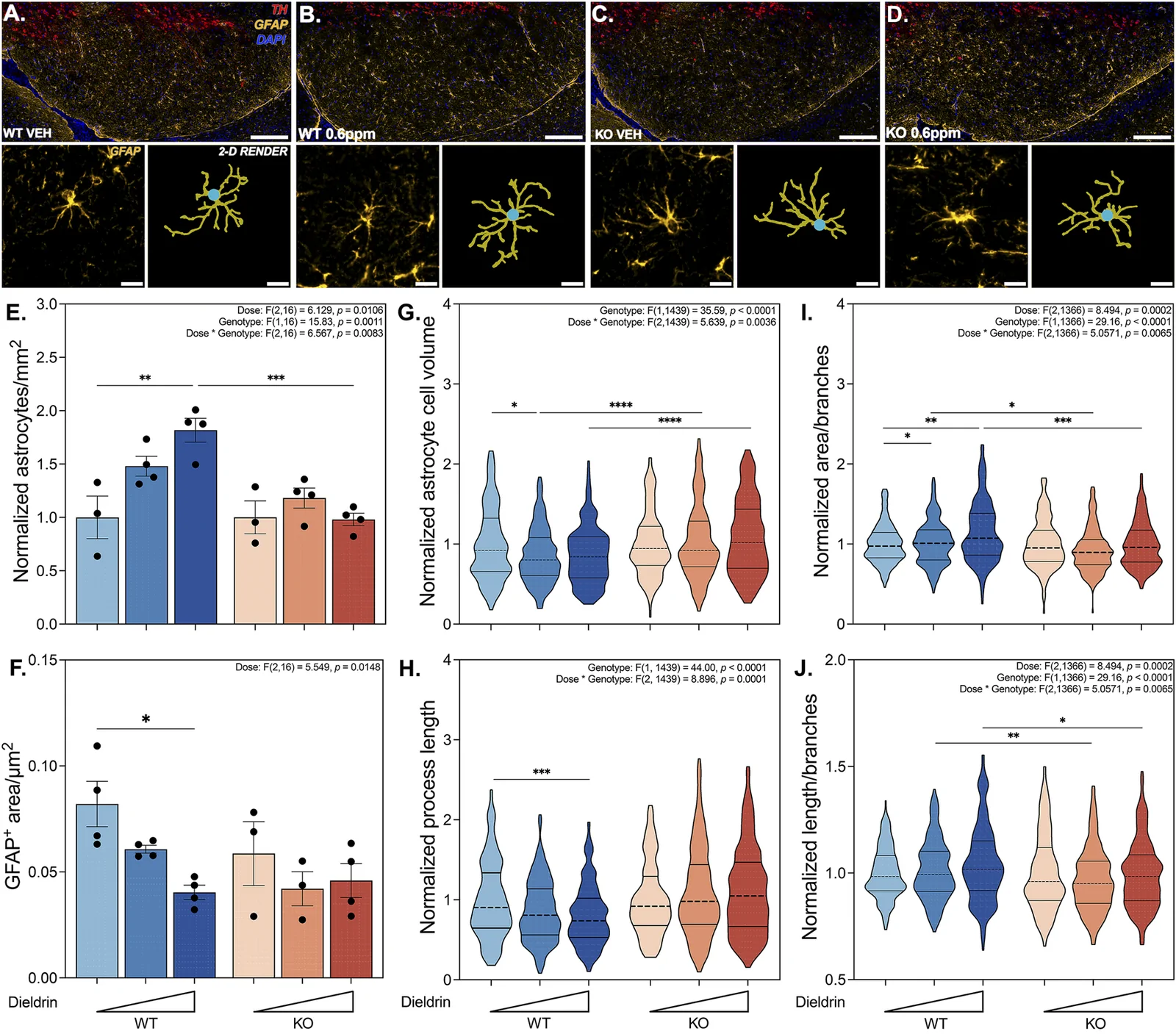
Dieldrin exposure alters density and morphology of wildtype SN astrocytes. Representative montage (scale bar 200 µm) and high magnification (scale bar 15 µm) images of the SN with immunofluorescently labeled astrocytes (GFAP, yellow), dopaminergic neurons (TH, red) and nuclei (DAPI, blue) as well as representative single-cell renders created in IMARIS **(A–D)**. Graphs depict the results of deep learning-based neural network assessment of astrocytic density **(E)** and % GFAP + area **(F)** within a region of interest encapsulating the SN, as well as GFAP^+^ cell volume **(G)**, summed process length **(H)**, volume/branch count **(I)**, and process length/branch count **(J)** at single-cell resolution. Parametric two-way ANOVAs with Bonferroni post-hoc correction were performed for data in panels **(E,F)**; bars represent mean ± SEM (n = 3–four mice/group). Nonparametric aligned rank transformed ANOVAs with Bonferroni post-hoc correction were performed for data in panels **(G–J)**; data are depicted as violin plot with median and quartiles (n = 170–280 astrocytes/mouse, 3–four mice/group). Statistically significant main and interaction effects denoted in the upper right-hand corner of each graph. Dieldrin doses utilized are 0 ppm, 0.2 ppm, and 0.6 ppm *p < 0.05, **p < 0.01, ***p < 0.001, ****p < 0.0001.

### 3.3 Dieldrin exposure results in reactivity-associated morphometric shifts in microglia

To further elucidate glial involvement in the neurological sequelae of dieldrin, quantitative digital pathology was applied to examine Iba1^+^ microglial density and morphology in the SN of male mice (Supplementary Figure S4; Figures 3A–D). A main effect of genotype was observed (p = 0.0037) for the density of Iba1^+^/DAPI^+^ cells, as well as a decrease in microglial density of HD KO mice relative to WT counterparts (37.11%, p = 0.0377). A reduction in Iba1^+^ cell volume of 25.5% (p < 0.0001) and 47.30% (p < 0.0001) was observed for WT LD and HD mice, respectively (Figure 3F). Microglia of KO HD mice displayed a comparable change (46.5%, p < 0.0001) in cell volume, with no alterations determined for KO LD mice. Total process length was reduced by 8.406% (p = 0.0005) in WT LD mice (Figure 3G). Examination of the number of branches and processes revealed a significant interaction by genotype and dose (branches: χ^2^ (2) = 123.06, p < 0.0001; processes: χ^2^ (2) = 42.16, p < 0.0001), as well as main effects for genotype (branches: χ^2^ (1) = 395.72, p < 0.0001; processes: χ^2^ (1) = 158.71, p < 0.0001), and dose (branches: χ^2^ (1) = 39.47, p < 0.001; processes: χ^2^ (1) = 19.158, p < 0.0001). As such, the effect of dose was examined separately for each genotype. WT microglia showed a decline of 41.40% (p < 0.0001) in the LD group, and 14.01% (p = 0.0400) in the HD group (Figure 3H). A similar response was noted for the number of processes in WT mice in the LD (22.93%, p < 0.0001) and HD (12.01%, p = 0.0029) conditions (Figure 3I). Dieldrin-dependent alterations were modulated for KO mice, with reductions in branches (21.71%, p = 0.0019) and processes (11.29%, p = 0.0265) observed in microglia of the HD, but not LD, condition.

**FIGURE 3:**
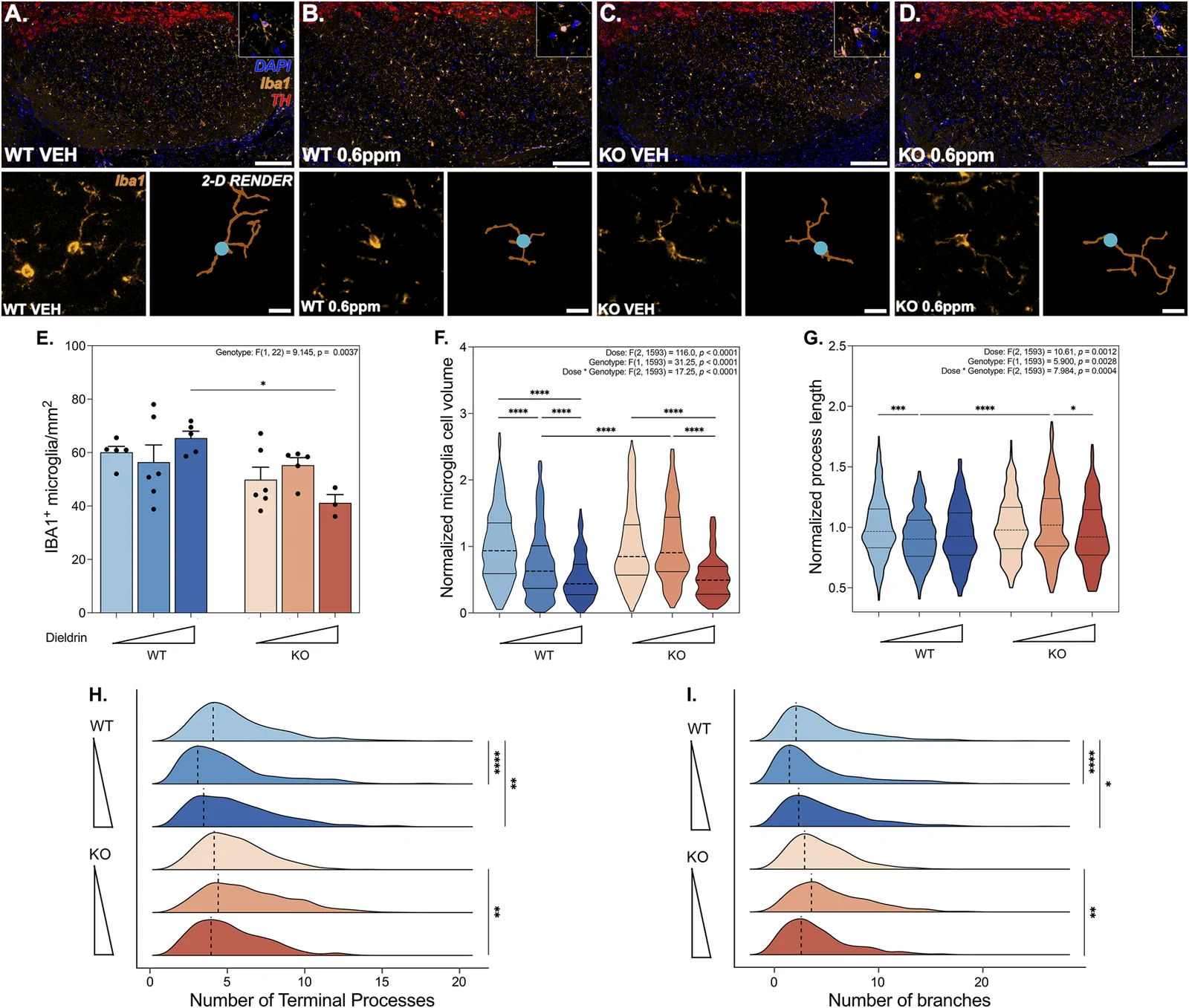
ALDH1/2 knockout moderates SN microglial reactivity following dieldrin exposure. Representative montage (scale bar 200 µm) and high-magnification (scale bar 15 µm) images of the SN with immunofluorescently labeled microglia (Iba1, orange), dopaminergic neurons (TH, red) and nuclei (DAPI, blue) as well as representative single-cell renders created in IMARIS **(A–D)**. Graphs depict the results of deep learning-based neural network assessment of microglial density **(E)**, microglial volume **(F)**, summed process length **(G)**, number of branches **(H)**, and number of terminal processes **(I)**. Parametric two-way ANOVA with Bonferroni post-hoc correction was performed for data in panel E; bars represent mean ± SEM (n = 3–6 mice/group). Nonparametric aligned rank transformed ANOVAs with Bonferroni post-hoc correction were performed for data in panels F and G; volcano plots represent median and quartiles (n = 153–358 microglia/mouse, 3–6 mice/group). Significant main and interaction effects denoted in the upper right-hand corner of graphs E-G. Negative binomial linear regressions with Bonferroni post-hoc correction were performed for graphs H and I; central measure of ridgeline plots depicts the mode. Dieldrin doses utilized are 0 ppm, 0.2 ppm, and 0.6 ppm. *p < 0.05, **p < 0.01, ***p < 0.001, ****p < 0.0001.

### 3.4 Microglial morphology clusters distinctly by genotype and dieldrin dose

Given the observed effects of genotype and dieldrin on glial density and morphometry, we sought to further probe differences in microglial phenotypes. UMAP plots were generated from morphological and cellular features queried from microglia located in the SN of male mice (Figure 4). Dimensional reduction analysis revealed distinct separation of microglia by genotype (Figure 4A). Further examination of genotype and treatment group demonstrate greater separation of WT microglia by dieldrin dose, as compared with KO microglia (Figure 4B). Immunoblotting experiments were performed to determine if this variation in microglial morphology is indicative of induction of inflammatory immune modulators (Supplementary Figure S5). A main effect of genotype was observed for both GFAP (p = 0.0207) and TNF-α (p = 0.0128) Supplementary Figures S5A,B). A main effect of dose (p = 0.0002) and genotype (p = 0.0322) was determined for IFN-γ, in addition to increases in WT LD (25.35%, p = 0.0019) and HD (57.65%, p = 0.0019) mice relative to KO counterparts (Supplementary Figure S5C). Overall, the data presented suggest sub-chronic dieldrin exposure elicits PD-like neurotoxicity accompanied by inflammatory gliosis in male WT mice, which appears to be protected against in Aldh1/2 KO mice (Figure 4C).

**FIGURE 4:**
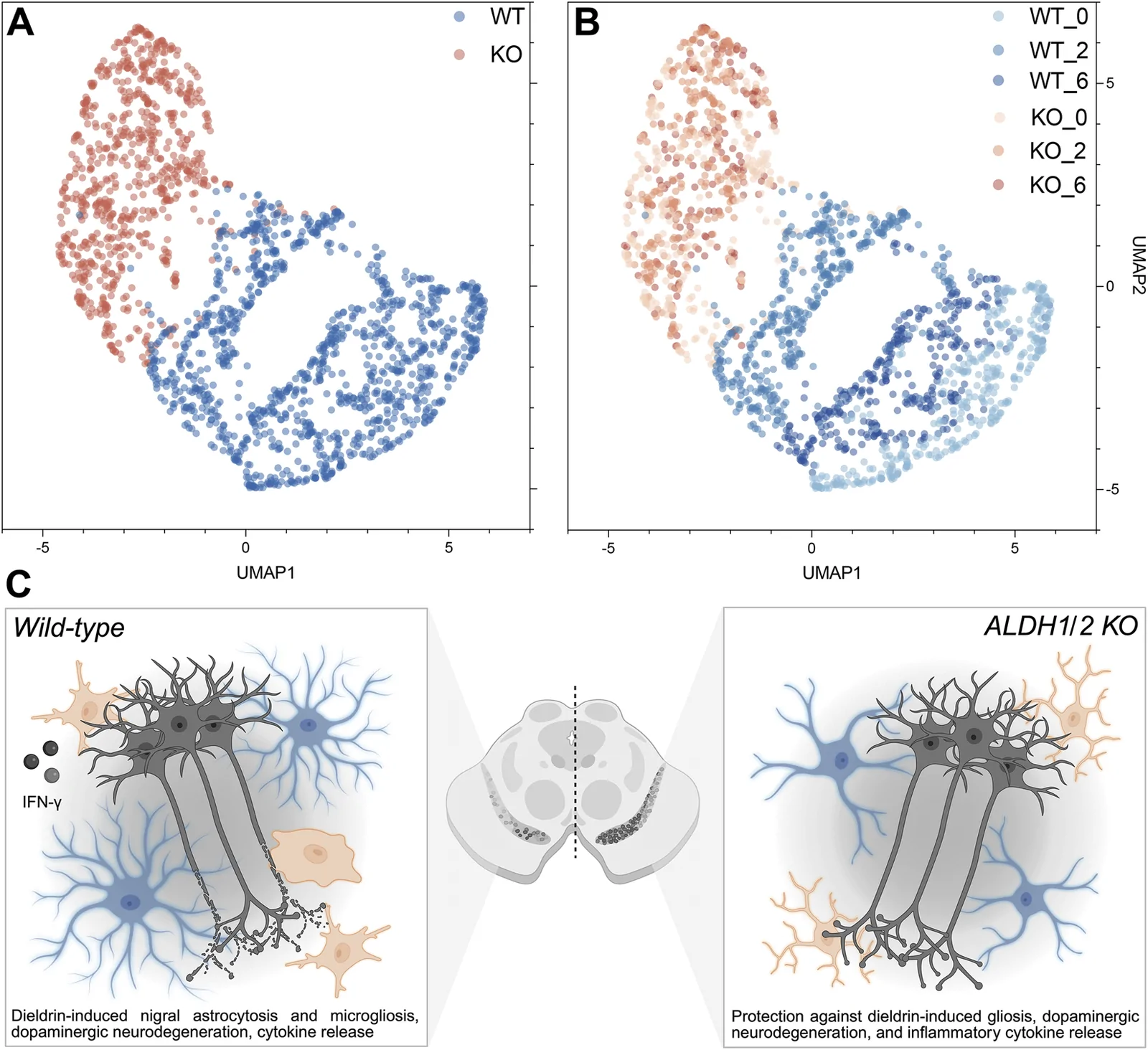
ALDH1/2 KO protects against dieldrin-induced PD-like neurotoxicity and inflammatory glial reactivity in mice. UMAP plots generated from 13 features (processes length, cell volume, number of branches, number of processes, convexivity, ellipticalness, form factor, perimeter, eccentricity, solidity, Iba1 intensity, DAPI intensity, and nuclear area) demonstrate variance in microglial morphometry by genotype **(A)** and dieldrin dose **(B)**. The findings from this study are summarized in panel **(C)** and suggest ALDH1/2 KO protects against dieldrin-induced PD-like glial activation and dopaminergic neurodegeneration.

## 4 Discussion

Exposure to environmental toxicants strongly correlates with PD risk, often described as an individual’s ‘exposome’ across the lifespan (Atterling Brolin et al., 2025).Dieldrin has been linked to nigrostriatal injury and increased risk for PD (Chhillar et al., 2013; Weisskopf et al., 2010; Fleming et al., 1994), and *in vitro* studies have shown dieldrin exposure decreases neuronal viability (Sanchez-Ramos et al., 1998). Mitochondrial dysfunction, ROS production, and impaired detoxification of reactive aldehydes have been identified as potential factors underlying the neurotoxicity of this pesticide (Allen et al., 2013; Kitazawa et al., 2003; Schmidt et al., 2017); however, these mechanisms are not fully characterized in this context. Together, this motivated the present assessment of glial reactivity and dopaminergic neurodegeneration in WT and Aldh1/2 KO mice sub-chronically exposed to dieldrin.

In the present study, deep-learning neural networks were utilized to perform an unbiased stereologic estimation of neuronal density within this nucleus. We observed exposure-induced degeneration of dopaminergic and total neurons in this brain region which were sex- and genotype-dependent (Figure 1). This is consistent with studies from Hatcher and colleagues (2007, 2008) which reported dieldrin-induced nigrostriatal DA injury in male mice (Hatcher et al., 2008; Hatcher et al., 2007). In the present study, we did not observe reductions in striatal dopaminergic terminal coverage (Supplementary Figure S3), which may be explained by compensatory reinnervation (Stanic et al., 2003). No overt neurodegeneration was observed in female mice, despite greater retention of dieldrin (Supplementary Table S1). Prior studies have attributed this discrepancy to male-specific variations in cytochrome P-450 isoenzymes which facilitates dieldrin clearance (in Toxicological Profile for Aldrin, 2022; Hutson, 1976; Klevay, 1970). This sex-specific neuropathology is consistent with studies reporting developmental dieldrin exposure to produce male-specific vulnerability to secondary insults (Gezer et al., 2020; Richardson et al., 2006), and is reflective of the sexual dimorphic incidence of human PD (Van Den Eeden et al., 2003; Adamson et al., 2022). Comparable female protection in toxicant models of PD has been attributed to the effects of endogenous estrogens (Adamson et al., 2022; De Miranda et al., 2019; Thadathil et al., 2021; Moreno et al., 2011). These data led us to examine morphologic changes in glial cells in the SN of male mice that may be associated with the dopaminergic neurotoxicity of dieldrin.

The role of astrocyte reactivity in dieldrin neurotoxicity has not been extensively examined, although developmental exposure has been suggested to prime inflammatory reactivity in dual-hit studies (Richardson et al., 2006). We have previously characterized astrocytic responses across toxicant-based models of PD (Rocha et al., 2022; Hammond et al., 2020) which share molecular consequences similar to dieldrin toxicity potentially contributing to this glial reactivity (Schmidt et al., 2017; Hatcher et al., 2007). To assess astrocytic states, we applied single-cell resolution morphometric analysis pipelines to examine parameters associated with activation (Hager et al., 2026). Dieldrin treatment resulted in dose-dependent increases in astrocytic density and reactive morphology in the SN of male WT mice, which were abrogated in Aldh1/2 KO counterparts (Figure 2D). Concurrently, we observed a decrease in % GFAP^+^ area in these WT mice, suggesting genotype-dependent differences in proliferation and morphometry (Figures 2E–J). Previous reports in models of neurodegeneration have characterized astrocytic atrophy which may be related to mitochondrial deficits (Kulijewicz-Nawrot et al., 2012; Olabarria et al., 2010; Popov et al., 2023). These data suggest sub-chronic dieldrin exposure is capable of eliciting dynamic astrocytic reactivity in male WT mice. As glial crosstalk is increasingly recognized as playing an important role in PD pathogenesis, we next examined microglial reactivity.

Morphologically reactive microglia have been reported in human PD and in models of this disease (Imamura et al., 2003; Kirkley et al., 2017; Bantle et al., 2019). Neural network-based morphometric analysis was therefore applied to Iba1^+^/DAPI^+^ cells in the SN to assess reactivity (Figures 3A–D). While the density of microglia was unchanged by dieldrin exposure, a significant main effect of genotype was identified (Figure 3F). Recent data suggest microglial counts alone are insufficient in assessing reactivity, particularly at a single time point (Hopperton et al., 2018; Sanchez-Guajardo et al., 2010; Green et al., 2022). Morphologic changes in microglia have been correlated with neuroinflammation in toxicant-based models of PD, providing a more reliable assessment of reactivity (Rocha et al., 2022; Hager et al., 2026; Hammond et al., 2020; Fernandez-Arjona et al., 2019). We report pronounced alterations in the morphology of microglia in dieldrin-exposed male WT mice consistent with an inflammatory, disease-associated phenotype (Figures 3F–I). These changes have been shown previously to be indicative of aberrant, pathologically reactive microglia (Heindl et al., 2018; Green and Rowe, 2024). Aldh1/2 KO moderated microglial responses to dieldrin (Figures 3F,H,I). Qualitative dimensional-reduction analysis revealed distinct separation of microglia by genotype and dose, suggesting Aldh1/2-dependent responses to dieldrin exposure (Figures 4A,B). Immunoblotting experiments further corroborate this, demonstrating robust WT-specific induction of inflammatory signaling proteins in male WT mice (Supplementary Figure S5) *In vitro* studies have observed superoxide production, a potent pro-inflammatory signal, in dieldrin-exposed microglial cultures (Mao et al., 2007). Additionally, evidence exists demonstrating ALDH2 deficiency to suppress phagocytic capacity and cytokine production in cultured microglial lines (Wang et al., 2024). Accordingly, Aldh1/2 KO appears to dampen microglial inflammatory activation in response to dieldrin.

Certain caveats must be noted in interpreting the findings of the present study. A previous report using this Aldh1/2 KO mouse showed age-dependent injury to the nigrostriatal system at 12–14 months (Wey et al., 2012). Other studies corroborate this age-dependent neurodegeneration and demonstrate that Aldh deficiency can exacerbate PD-relevant neurotoxic insults (Martinez et al., 2023; Anvret et al., 2012; Li et al., 2025). As this study employed 6-month-old mice, alternative aldehyde-clearance pathways may have compensated for this deficiency. Aldehyde reductase (AR) and aldo-keto reductases constitute minor aldehyde-clearing pathways (Juarez Olguin et al., 2016; Meiser et al., 2013), and prior investigation has shown greater involvement of AR in this Aldh1/2 KO mice (Goldstein et al., 2013). Additionally, dieldrin is eliminated more rapidly by rodents than humans (in Toxicological Profile for Aldrin, 2022; Hunter et al., 1969; World Health, 1993; Organization, 1989). Although dieldrin levels reported here are consistent with human-relevant exposures (2 ng/g – 505 ng/g) (Corrigan et al., 2000; Fleming et al., 1994; Corrigan et al., 1996), there may exist species-specific susceptibility toward injury. The sample size employed in this study also has the potential to mask moderate changes, particularly in cases of three-factor analyses. Despite these considerations, our data highlight compelling evidence of a protective role of ALDH ablation in the context of dieldrin-induced neurotoxicity.

In the present study, we investigated the consequences of Aldh1/2 deficiency on PD-relevant neuropathology in a murine pesticide exposure model. These data demonstrate sub-chronic dieldrin administration via diet resulted in genotype- and sex-dependent neuronal loss in the SN. This report is the first of our knowledge to characterize disease-associated morphometric changes in glial phenotypes following exposure to dieldrin. Aldh1/2 KO abrogated the selective neurodegeneration and pro-inflammatory changes to glial reactivity observed in this study. Together, these findings support the idea that ALDH enzymes may play a role in the pathogenesis of dieldrin-induced parkinsonism. Future work is necessary to determine the mechanism by which Aldh1/2 ablation impacts glial-mediated inflammation and potential functional behavioral deficits, as well as the temporal relationship of this phenomenon with selective nigrostriatal dopaminergic neurotoxicity.

## Data Availability

The raw data supporting the conclusions of this article will be made available by the authors, without undue reservation.
