# Supplementary material for: ALDH1/2 deficiency modulates dieldrin-induced dopaminergic neurodegeneration and reactive glial morphometry in male mice
Source: Front Toxicol. 2026 Sep 15;8:1888799. doi: 10.3389/ftox.2026.1888799 (PMC13619124; doi:10.3389/ftox.2026.1888799)
Supplement: Supplementary file 1 [file DataSheet1.docx]

Supplementary Materials

**Supplementary Table 1**. Dieldrin concentration measured in striatal tissue punches (n = 4 – 5 samples/group) via gas chromatograph coupled with a single quadrupole mass spectrometer. ND = samples below limit of detection.

| **Dieldrin (ng/g wet tissue)** | | | | | | | |
| --- | --- | --- | --- | --- | --- | --- | --- |
| **Male Wild Type** | | **Male Knockout** | | **Female Wild Type** | | **Female Knockout** | |
| **0.2ppm** | **0.6ppm** | **0.2ppm** | **0.6ppm** | **0.2ppm** | **0.6ppm** | **0.2ppm** | **0.6ppm** |
| 83.50 | 119.15 | 74.58 | 135.11 | 84.53 | 253.09 | 66.04 | 114.84 |
| 74.53 | 109.87 | ND | 135.84 | 75.42 | 222.49 | 64.90 | 91.63 |
| ND | 85.85 | ND | 121.35 | 70.38 | 173.84 | 78.87 | 155.40 |
| ND | 99.90 | ND | 129.04 | 64.73 | 123.12 | ND | 111.52 |
| ND | 109.29 |  |  | 91.38 | 153.24 |  | 146.83 |

**Supplementary Figure 1**. Bar graphs depicting baseline weights (A), weights after the six-week study duration (B), and change in body weight from baseline for male (C) and female (D) mice (n = 8-10 mice/group). Data are depicted as mean ± SEM. *, **, *** represent p-values < 0.05, 0.01, and 0.001, respectively.

**Supplementary Figure 2**. Representative brightfield montage images (scale bar = 100µm) and high-magnification insets (scale bar = 15µm) of hematoxylin and eosin-stained sections, as well as masked images depicting neural networks trained to identify healthy (blue) and pyknotic nuclei (red).

**Supplementary Figure 3**. Bar graph depicting the intensity (arbitrary units; AU) of TH+ projections in the dorsal striatum quantified against background tissue intensity. Statistical analysis was performed using a parametric three-way ANOVA with Bonferroni post-hoc correction. Significant main and interaction effects are denoted in the upper right-hand corner of the graph. Data are represented as mean ± SEM (n = 5-6 animals/group).

**Supplementary Figure 4**. Representative montage image (A) of the substantia nigra (SN) immunofluorescently labeled for cellular nuclei (DAPI, blue), microglia (Iba1, orange), dopaminergic neurons (TH, cyan). High-magnification insets (B) depict the a representative microglia in this region (lefta) and the skeletonization mask applied to quantify morphometric parameters (right).

**Supplementary Figure 5**. Bar graphs depicting quantification of GFAP (A), TNF-α (B), and IFN-γ (C) via immunoblotting in striatal tissue homogenate obtained from male mice (n = 3 – 5 mice/group). Parametric two-way ANOVA with Bonferroni post-hoc correction was performed; data are depicted as mean ± SEM. *, ** depict p-values < 0.05 and 0.01, respectively.
